# A Cluster-Based Approach for the Discovery of Copy Number Variations From Next-Generation Sequencing Data

**DOI:** 10.3389/fgene.2021.699510

**Published:** 2021-06-28

**Authors:** Guojun Liu, Junying Zhang

**Affiliations:** School of Computer Science and Technology, Xidian University, Xi’an, China

**Keywords:** next-generation sequencing, copy number variation, clustering algorithm, abnormal score, Tukey’s fences

## Abstract

The next-generation sequencing technology offers a wealth of data resources for the detection of copy number variations (CNVs) at a high resolution. However, it is still challenging to correctly detect CNVs of different lengths. It is necessary to develop new CNV detection tools to meet this demand. In this work, we propose a new CNV detection method, called CBCNV, for the detection of CNVs of different lengths from whole genome sequencing data. CBCNV uses a clustering algorithm to divide the read depth segment profile, and assigns an abnormal score to each read depth segment. Based on the abnormal score profile, Tukey’s fences method is adopted in CBCNV to forecast CNVs. The performance of the proposed method is evaluated on simulated data sets, and is compared with those of several existing methods. The experimental results prove that the performance of CBCNV is better than those of several existing methods. The proposed method is further tested and verified on real data sets, and the experimental results are found to be consistent with the simulation results. Therefore, the proposed method can be expected to become a routine tool in the analysis of CNVs from tumor-normal matched samples.

## Introduction

The copy number variation (CNV) of DNA fragments has been widely recognized as a major type of structural variations, and can cause the amplification or deletion of DNA fragments, the lengths of which are greater than 1 kbp in the human genome (Freeman et al., 2006). Some CNVs, called germline CNVs, are also present in normal tissues of the human body; these generally originate from family inheritance, and can cause cancers and diseases (Kuiper et al., 2010; Krepischi et al., 2012). The CNVs in tumor tissue are generally called somatic CNVs, which are acquired CNVs, and cause tumor formation by oncogene and tumor suppressor gene mutations (Stratton et al., 2009; Beroukhim et al., 2010; Pei et al., 2020). Many experimental studies have proven that CNVs can change the doses of genes and lead to the reorganization of chromosome structure (Sharp et al., 2005; Magi et al., 2017; Pei et al., 2021b), and makes an important contribution to the occurrence and formation of tumors and various disorders (Pei et al., 2021a). For example, it can cause schizophrenia and autism disorders in humans (Sebat et al., 2007; Cook and Scherer, 2008; Stone et al., 2008). Some studies have shown that CNVs are related to cancer, such as breast and ovarian cancer (Tchatchou and Burwinkel, 2008; Adam and David, 2009; Malek et al., 2011). In practical applications, there is a strong requirement to capture CNVs of various range lengths, which requires the developed tools to have higher resolution and better robustness than previously developed tools to reduce the false positive rate of test results. Therefore, it is still a difficult task to effectively detect CNVs of different lengths.

Compared with traditional (array-based) detection methods (Carter, 2007; Buysse et al., 2009), the detection cost has been greatly reduced and resolution has reached the base-pair level with the emergence of next-generation sequencing technology. In recent years, most related tools for CNV detection using next-generation sequencing data have been developed based on paired-end mapping (PEM) (Korbel et al., 2007) and depth of coverage (DOC) (Yoon et al., 2009) strategies. The basic concept of PEM-based methods is that the insertion size of aligned paired-end reads is significantly different from the insertion size preset by the laboratory (Medvedev et al., 2009). While PEM-based methods can detect amplification, deletion, insertion, translocation, etc., they can only identify those insertion variants whose lengths are less than the preset insertion length. The basic concept of DOC-based methods is that the number of reads aligned to each position of the reference genome is proportional to the number of copies corresponding to that position (Yoon et al., 2009). In principle, DOC-based methods can detect CNVs of various lengths. However, in practical applications, they are more suitable for the detection of long CNVs, and cannot accurately detect the boundaries of the CNVs.

Generally, DOC-based methods require the input of tumor-normal matched samples to detect the tumor genome and effectively capture CNVs. The workflow of this type of method is: (1) input tumor-normal matched samples; (2) obtain read count profiles with SAMtools (Li et al., 2009); (3) bin read count profiles (Chiang et al., 2009) and generate read depth profiles; (4) use the joint read depth information of the tumor-normal matched samples to build a statistical model; (5) choose a suitable threshold to predict CNVs. It is generally believed that the deviation caused by sequencing is consistent in the same areas of the two samples. Therefore, DOC-based methods use the read depth ratio information to eliminate these deviations (GC content and mappability biases) (Bentley et al., 2008; Chiang et al., 2009). Some well-known methods have been developed to detect CNVs from tumor-normal matched samples, including BIC-seq2 (Xi et al., 2016), SeqCNV (Chen et al., 2017), and CNVkit (Talevich et al., 2016). BIC-seq2 preprocesses the sequenced reads, including by calibrating the GC content bias, removing mappability bias, and normalizing reads at the nucleic acid level. Based on the preprocessed data, the segmentation procedure is executed using the bayesian information criterion, by which CNVs are forecasted. It is not sensitive to the detection of short CNVs. SeqCNV extracts the read depth information of the tumor-normal matched samples to build a maximum penalized likelihood estimation model to predict CNVs. It detects a small number of CNVs, most of which are the gain areas and true positives, and its detection is more conservative than that of BIC-seq2. It has a long running time and is not suitable for testing samples with long CNVs. CNVkit is a software toolkit that extracts the information of on- and off-target sequenced reads. It adopts a rolling median method to normalize the GC content bias, mappability bias, and target density bias, and to reduce the impact on the true copy number status. CNVkit detects the CNVs, many of which are deletion regions. However, it is not sensitive to the detection of short CNVs.

In consideration of the limitations of the existing methods, in this study, a new tumor-normal matched sample-based CNV detection method, called CBCNV (cluster-based approach for CNV detection), is proposed for the prediction of CNVs using whole-genome sequencing data. CBCNV extracts the read count profiles of tumor-normal matched samples with SAMtools (Li et al., 2009). The preprocessing program is executed on the read count profiles, which can yield the read depth segment profiles, the dimensions of which are transformed into two-dimensional space. CBCNV adopts the k-means algorithm to cluster the preprocessed read depth segment profiles (Hartigan and Wong, 1979), which can yield clusters of different sizes. The clusters are sorted from largest to smallest according to the number of elements in each cluster. Then, by setting a boundary threshold, these clusters are divided into large and small clusters. Based on the above definition, CBCNV assigns a cluster-based abnormal score for each read depth segment. Using the cluster-based abnormal score profiles, Tukey’s fences method is employed to announce candidate CNVs (Zijlstra et al., 2007). The performance of the proposed method is verified using simulated and real data sets, and is compared with several existing CNV detection methods. The experimental results show that the performance of CBCNV is better than several other comparison methods, especially for low-purity samples. In addition, CBCNV is also found to detect some biologically meaningful CNVs, which can provide some valuable reference information for assistance with clinical diagnosis and targeted drug research.

The remainder of this article is organized as follows. Section “Materials and Methods” includes the workflow of CBCNV, data preprocessing, the calculation of cluster-based abnormal score, and the prediction of CNVs. In section “Results,” simulation and real experiments are designed, and the experimental results are analyzed and discussed. Section “Discussion and Conclusion” summarizes this research and puts forward ideas for future work.

## Materials and Methods

### Overview of CBCNV

**FIGURE 1:**
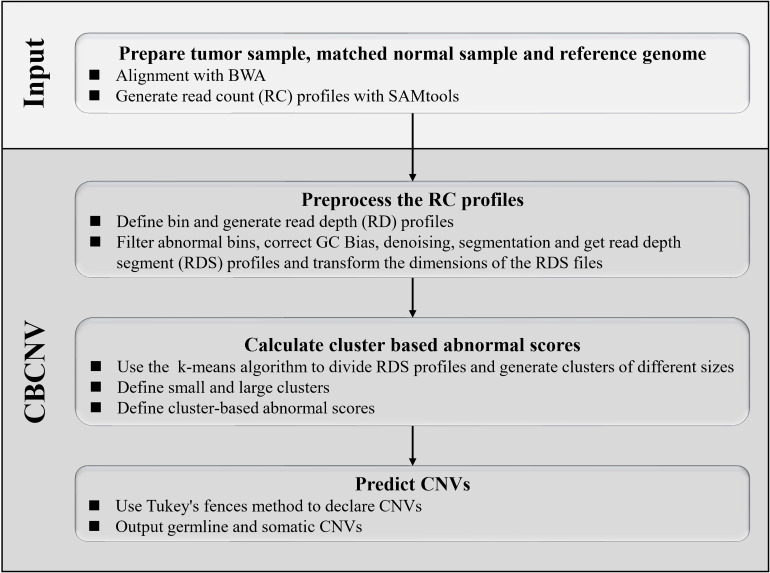
Overview of the workflow of CBCNV. It is mainly composed of four steps, which includes preparing input files, preprocessing read count profiles, calculating cluster-based abnormal scores, and recording CNVs.

CBCNV is a DOC-based approach that is suitable for the detection of tumor-normal matched samples, and can identify somatic CNVs and germline CNVs from whole-genome sequencing data. The pipeline of CBCNV is described in detail in Figure 1. The sequenced tumor-normal matched samples that are composed of a large number of sequenced reads are compared to the reference genome using the BWA tool (Li and Durbin, 2010). Then, the read count profiles of the tumor-normal matched samples are generated with SAMtools (Li et al., 2009). Based on the read count profiles, the following four steps are conducted for CBCNV to complete CNV detection. The first step involves defining the bin, dividing the reference genome into continuous and non-overlapping regions according to the bin size, and generating the read depth profiles. In the second step, the abnormal bins are removed, and the GC content bias is corrected. The read depth profiles are denoised and segmented to generate the read depth segment profiles. The dimensions of the read depth segment profiles are converted from one-dimensional to two-dimensional space. In the third step, the preprocessed read depth segment profiles are clustered via the k-means method to form clusters of different sizes. A boundary value is set to divide large and small clusters. The cluster-based abnormal score is defined based on the following two situations (He et al., 2003): (1) if a read depth segment belongs to a small cluster, the cluster-based abnormal score is defined as the distance between the read depth segment and the center of the large cluster that is the closest to it; (2) if a read depth segment belongs to a large cluster, the cluster-based abnormal score is defined as the distance between the read depth segment and the center of the large cluster. Finally, in the fourth step, Tukey’s fences method is employed to predict CNVs (Zijlstra et al., 2007). The CBCNV software is developed based on the R and Python languages (Zhao et al., 2019). Its source code is public, and can be downloaded from https://github.com/gj-123/CBCNV/releases, where users can easily install and use the software according to the instructions.

### Data Preprocessing

The sequenced reads are aligned to the reference genome with BWA (Li and Durbin, 2010), and the read count profiles are generated by SAMtools (Li et al., 2009). The reference genome is composed of five types of positions (“A”, “T”, “G”, “C”, and “N”). Here, “N” indicates the base positions that cannot be determined during the sequencing process. The sequenced reads cannot be matched to the “N” positions, which are often mistaken for CNV deletion regions. To obtain reasonable read count profiles, a binning strategy is adopted to deal with the “N” positions (Yuan et al., 2018). The read count profiles are divided into continuous and non-overlapping areas according to the bin size. The bins that contain the “N” positions are treated as abnormal bins and filtered out. The mean read count value of each bin is calculated to obtain the read depth profiles. Based on the above processing, Eq. (1) is used to deal with GC content bias (Yoon et al., 2009):

(1) $$RD_{i}^{\prime }=RD_{i}\cdot \frac{RD_{m}}{RD_{gc}},$$

where *RD*_*i*_ and $RD_{i}^{\prime }$ represent the original and revised read depth values of the i-th bin, respectively, *RD*_*m*_ represents the mean value of the read depth of all bins, and *RD*_*gc*_ represents the mean read depth value of the bins, the GC content of which is equal to that of the i-th bin. Sequencing errors and various deviations will lead to a substantial amount of noise in the read depth data, and ultimately false test results. Thus, noise reduction is a necessary step in CNV detection. The fused lasso regression method is adopted to smooth the read depth profile (Tibshirani and Wang, 2008). This method effectively considers the copy number relationship between adjacent read depth signals, which allows a reasonable read depth segment profile to be obtained. Based on the denoised read depth segment profile, Eqs. (2–5) (Li Y. et al., 2019; Liu et al., 2020) are used to transform its dimensions.

(2) CN=CNnorm⋅RDSiRDSm  1≤i≤|RDS|

(3) RDSR=RDSiRDSm   1≤i≤|RDS|

(4) RDSD={∑j=i+1i+L|RDSRi-RDSRj|L  i=1,5≤L≤20∑j=1i+L|RDSRi-RDSRj|i-1+L      1<i≤L,5≤L≤20∑j=i-Li+L|RDSRi-RDSRj|2L  L<i≤|RDS|-L,5≤L≤20∑j=i-L|RDS|-1|RDSRi-RDSRj|L+|RDS|-i-1|RDS|-L<i≤|RDS|-1,5≤L≤20∑j=i-Li-1|RDSRi-RDSRj|L  i=|RDS|,5≤L≤20

(5) $$RDS^{\prime }=\left\{CN,RDSD\right\}$$

In Eq. (2), *CN*_*norm*_ represents the normal copy number, and its value is equal to 2. Additionally, *RDS*_*i*_ represents the value of the i-th read depth segment, *RDS*_*m*_ represents the mean across all the read depth segments, and CN represents the set of copy number, which is composed of the copy number of all read depth segments. |*RDS*| represents the number of elements in the read depth segment set. In Eq. (3), *RDSR* represents a set that is composed of the ratio between *RDS*_*i*_ and *RDS*_*m*_. In Eq. (4), L represents the number of left and right neighbors of the i-th element of *RDSR*, and is set to 10 by default. |*RDSR*_*i*_−*RDSR*_*j*_| represents the absolute value of the difference between *RDSR*_*i*_ and *RDSR*_*j*_, and *RDSD* represents the set of differences of each element in *RDSR*. In Eq. (5), *RDS*′ represents a two-dimensional data set, which is composed of CN and *RDSD*. This processing step provides two perspectives to observe read depth segments. The first dimension can approximately reflect the copy number status for each read depth segment, which provides a longitudinal and global perspective to observe the trend of copy number changes. The second dimension indirectly reflects the difference between a read depth segment and its surrounding read depth segments, which provides a horizontal and partial perspective to illustrate the relevance of the copy number status of each read depth segment. Moreover, this processing step provides a valid data set for the calculation of cluster-based abnormal scores, which is elaborated in the next subsection.

### Calculation of Cluster-Based Abnormal Scores

Based on the *RDS*′ profile, a cluster-based abnormal score is calculated for each read depth segment. Here, each element of *RDS*′ is regarded as an object *O*. The cluster-based abnormal score is designed based on the concept of CBLOF (He et al., 2003), and is different from the traditional tumor-normal matched samples based CNV detection methods, which utilize read depth information to fit a statistical model and set a threshold to predict CNVs. The cluster-based abnormal score reflects the isolation degree of the local small cluster relative to the large cluster around it, as well as the deviation degree of each object in the large cluster relative to its cluster center, which indirectly reflects the abnormal degree of the copy number of each object. If the cluster-based abnormal score of an object is higher than those of most objects, it is likely a CNV. To further calculate the cluster-based abnormal scores, the definition is subsequently introduced in detail. First, the k-means algorithm is executed on the data set *RDS*′, and can divide the data set to form clusters of different sizes. Equation (6) is used to describe the clustering result:

(6) $$RDSC=\left\{RDSC_{1},RDSC_{2},\cdots ,RDSC_{k-1,}RDSC_{k}\right\},$$

$$RDSC_{i}\cap RDSC_{j}=\emptyset ,1\leq i\leq k,1\leq j\leq k,i\neq j,$$

where RDSC represents a set of k clusters. Second, based on the first step, RDSC is divided into large and small clusters (He et al., 2003), as given by Eqs. (7–11).

(7) $$RDSC^{\prime }=\left\{RDSC_{1}^{\prime },RDSC_{2}^{\prime },\cdots ,RDSC_{k-1}^{\prime },RDSC_{k}^{\prime }\right\}$$

(8) $$\left|RDSC_{1}^{\prime }\right|+\left|RDSC_{2}^{\prime }\right|+\cdots +\left|RDSC_{\theta }^{\prime }\right|\geq \left|RDSC^{\prime }\right|\cdot x$$

(9) $$\frac{\left|RDSC_{\theta }^{\prime }\right|}{\left|RDSC_{\theta +1}^{\prime }\right|}\geq y$$

(10) $$LRDSC^{\prime }=\left\{RDSC_{i}^{\prime }|1\leq i\leq \theta \right\}$$

(11) $$SRDSC^{\prime }=\left\{RDSC_{j}^{\prime }|\theta <j\leq k\right\}$$

$$RDSC_{1}^{\prime }|\geq |RDSC_{2}^{\prime }|\geq \cdots \geq |RDSC_{k-1}^{\prime }|\geq |RDSC_{k}^{\prime }|,$$

1≤i≤k,1≤j≤k,i≠j

In Eq. (7), *RDSC*′ represents the sorted cluster set RDSC, which is sorted in descending order. In Eq. (8), | ^∗^| represents the number of elements in a cluster, θ represents the boundary threshold of large and small clusters, and x represents a ratio between the total number of objects in the large cluster and the total number of objects in all clusters. The definition of the Eq. (8) is based on the consideration that most objects in *RDSC*′ are not CNVs. Thus, the clusters that contain most of the objects are considered large clusters. Eq. (9) signifies that the size of a large cluster is at least y times the size of a small cluster, and describes the difference in size between the smallest large cluster and the largest small cluster. In Eq. (10), *LRDSC*′ represents the set of large clusters. In Eq. (11), *SRDSC*′ represents the set of small clusters. Finally, based on the preceding definitions, Eq. (12) is constructed to describe the cluster-based abnormal score.

(12) $$\begin{array}{c} CBAS\left(O\right)=\\ \left\{ \begin{array}{c} min\left(dist\left(O,RDSC_{i}^{\prime }\right)\right)O\in RDSC_{j}^{\prime },RDSC_{i}^{\prime }\in LRDSC^{\prime },RDSC_{j}^{\prime }\in \\ SRDSC^{\prime },1\leq i\leq \theta ,\theta <j\leq k\\ dist\left(O,RDSC_{i}^{\prime }\right)O\in RDSC_{i}^{\prime },RDSC_{i}^{\prime }\in LRDSC^{\prime },1\leq i\leq \theta \end{array}\right. \end{array}$$

In Eq. (12), CBAS (*O*) represents the cluster-based abnormal score of object *O*, which is defined in two cases: (1) if the object *O* originates from a small cluster, the distance between *O* and the center of the closest large cluster is considered as the cluster-based abnormal score of *O*; (2) if the object *O* originates from a large cluster, the distance between *O* and the center of the large cluster is considered as the cluster-based abnormal score of *O*.

### Predicting CNVs

Based on the cluster-based abnormal score profiles, the abnormal objects must be identified. For this step, the traditional methods analyze the abnormality of each object, and the users directly select an appropriate threshold to cut off the abnormal objects according to the application scenario. In the proposed method, Tukey’s fences method is adopted to determine the abnormal objects. The prediction of abnormal objects consists of the following five steps. (1) the cluster-based abnormal scores of all objects are sorted from smallest to largest. (2) Eq. (13) is defined to evaluate an extreme outer limit:

(13) $$T=CBAS_{Q_{3}}+w\cdot \left(CBAS_{Q_{3}}-CBAS_{Q_{1}}\right),$$

where T represents the upper limit of fences, w represents an abnormal weight, *CBAS*_*Q_1*_ represents the cluster-based abnormal score of the lower quartile, and *CBAS*_*Q_3*_ represents the cluster-based abnormal score of the upper quartile. (3) the basic notion of judging abnormal objects is that the higher the cluster-based abnormal score of an object, the more likely it is to be a CNV. Here, T is used as the baseline to identify abnormal objects. If the cluster-based abnormal score of an object is greater than T, it is considered to be a CNV. If the cluster-based abnormal score of an object is less than or equal to T, it is considered to be a normal area. (4) after the candidate CNVs are determined, their mutation modes (gain or loss) are determined. If the read depth value of a CNV area is greater than or equal to the mean read depth value of all normal areas, it is considered to be a gain area. If the read depth value of a CNV area is less than the mean read depth value of all normal areas, it is considered to be a loss area. (5) finally, somatic CNVs and germline CNVs are further identified. A germline CNV is a genetic variation that may originate from an individual’s parents or family. If a CNV exists in both the tumor-normal matched samples, it is regarded as a germline CNV.

### Parameter Setting of CBCNV

To effectively use CBCNV, it is necessary to further explain the settings of related parameters, which include the bin size, the number of neighbors (L), the number of clusters (k), and the ratios of large clusters (x), multiples (y), and abnormal weight (w). In this study, the bin size and L are set to 2,000 bp and 10 by default, respectively. Additionally, the values of k, x, and y are set to 5, 0.9, and 5, respectively, which are adopted by referencing published article (He et al., 2003). In Tukey’s fences method, w is generally set to 1.5 (Zijlstra et al., 2007). In the proposed method, w is set to 1.5 as the default value. The settings of these default parameter values in the proposed method were determined according to experience and related methods. Users can also adjust these parameters according to their actual needs and application scenarios.

## Results

It is necessary to design a reasonable experimental plan to verify the effectiveness and reliability of the proposed method. Aiming at this point, simulation and real experiments were conducted. A simulation experiment is an effective and objective evaluation strategy, which can provide a comparison criterion to quantify the performance of the proposed method. In the simulation experiment, three popular published algorithms (BIC-seq2 (Xi et al., 2016), SeqCNV (Chen et al., 2017), and CNVkit (Talevich et al., 2016)) that can be used to effectively detect tumor-normal matched samples were selected for comparison with CBCNV. The performances of these methods are evaluated from three perspectives. First, the sensitivity and false discovery rate (FDR) of the four methods are evaluated at six CNV length levels. Then, the sensitivity and FDR of each method in the CNV gain and loss regions are analyzed and discussed. Finally, three indicators (recall, precision, and F1-score) are used to comprehensively evaluate the performance of each method. In real data applications, the proposed algorithm was used to detect two pairs of matched breast cancer whole-genome sequencing samples. Because the ground truths of the real data sets are unknown, the number of overlapping events and number of predicted events are adopted to evaluate the performance of each method. To further verify the performance of the proposed method, we use overlapping density score method to quantify performance of each method. The experimental results demonstrate that CBCNV is powerful CNV detection tools.

### Application of Simulation Data

Many CNV simulation softwares have been developed and applied to generate next-generation sequencing data. In this study, IntSIM software was selected to generate simulation data sets (Yuan et al., 2017). Before its use, some settings were conducted: (1) the reference genome was prepared; (2) the tumor purity (TP) and sequencing coverage (SC) were set; (3) the number of repetitions of the sample under the configuration of each group was selected. Chromosome 21 of hg19 was entered into the software as a reference genome. The tumor purity was set to 0.2 and 0.3, and sequencing coverage was set to 10× to generate simulated data sets of different configurations, in which 50 samples were generated. Each sample was embedded with 22 regions of variation, which were composed of 12 gains and 10 losses (four heterogeneous losses and six homogeneous losses). The length of the CNV regions ranged from 2 to 100 kb. To fairly evaluate the performance of each method, the default parameters were used for all methods to detect each set of data.

**FIGURE 2:**
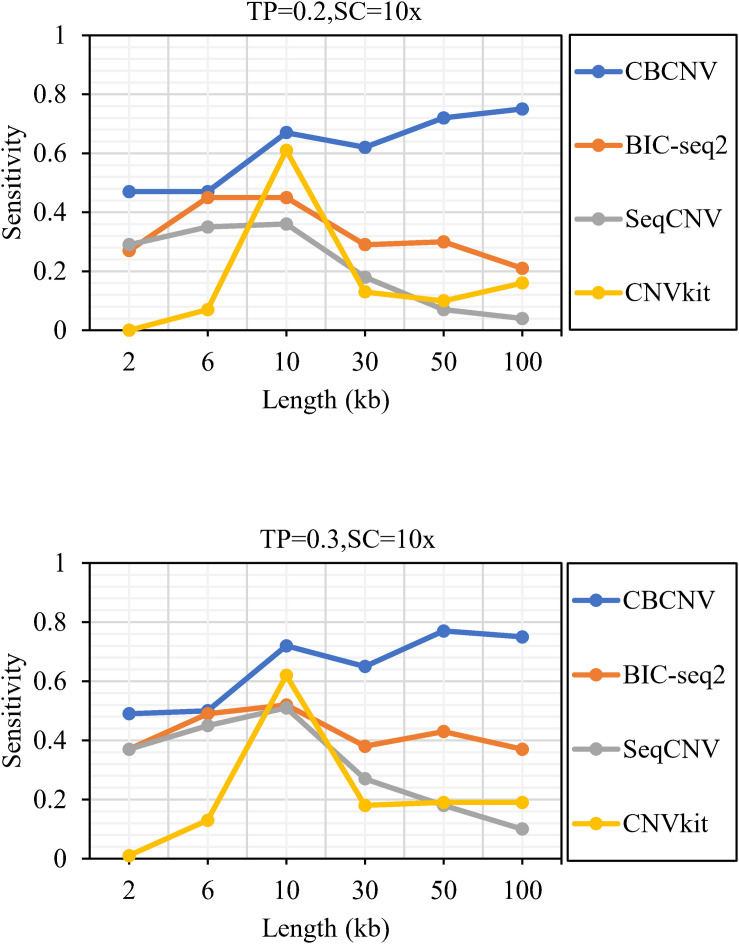
The sensitivity of four methods at the six CNV length levels under two sets of simulation configurations.

**FIGURE 3:**
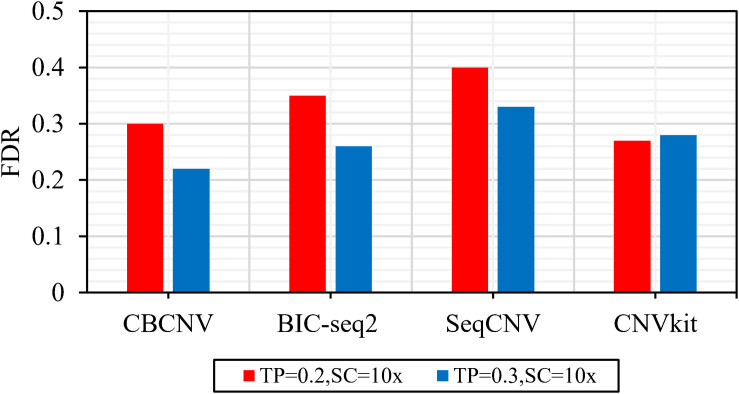
The FDR of each method at the six CNV length levels under two sets of simulation samples.

Figure 2 describes the sensitivity of the four methods for the respective detection of CNVs with lengths of 2, 6, 10, 30, 50, and 100 kb under two different configurations, respectively. Two performance indicators (sensitivity and FDR) are adopted to evaluate the resolution of each method. Sensitivity is defined as the value of the number of CNVs correctly detected by a tool divided by the total number of CNVs recorded by the ground truth file. FDR is defined as the value of the number of false positives detected by a tool divided by the total number of CNVs detected by the tool. If a detected event overlaps with the ground truth file by more than 50%, it is considered as a candidate CNV (Hormozdiari et al., 2009). From the figure, it is evident that the sensitivity of each method increased with the increase in tumor purity from 0.2 to 0.3. This demonstrates that tumor purity is one of the key factors that affect CNV detection. In contrast, long CNVs were more easily detected by each method than short CNVs. CBCNV achieved the best sensitivity for all CNV length levels, and BIC-seq2 achieved better sensitivity than the other two methods (SeqCNV and CNVkit) at most CNV length levels. SeqCNV achieved the lowest sensitivity in the cases of CNVs with lengths of 50 and 100 kb, which indicates that it is not sensitive enough to detect long CNVs. CNVkit achieved the lowest sensitivity in the cases of CNVs with lengths of 2 and 6 kb, which indicates that it is not sensitive enough to detect short CNVs. Figure 3 presents the FDR of each method at the six CNV length levels under two different configurations. In the case of tumor purity = 0.2, CNVkit performed the best in terms of FDR, followed by CBCNV, BIC-seq2 and SeqCNV. Although CNVkit achieved the best FDR, it had the lowest sensitivity. In the case of tumor purity = 0.3, CBCNV performed excellently in terms of FDR, followed by BIC-seq2, CNVkit, and SeqCNV. Considering the two indicators together, CBCNV achieved the best tradeoff between sensitivity and FDR, followed by BIC-seq2, SeqCNV, and CNVkit. Via the preceding analysis and discussion, it can be concluded that CBCNV can detect more CNVs with fewer false positives than the other three methods.

**FIGURE 4:**
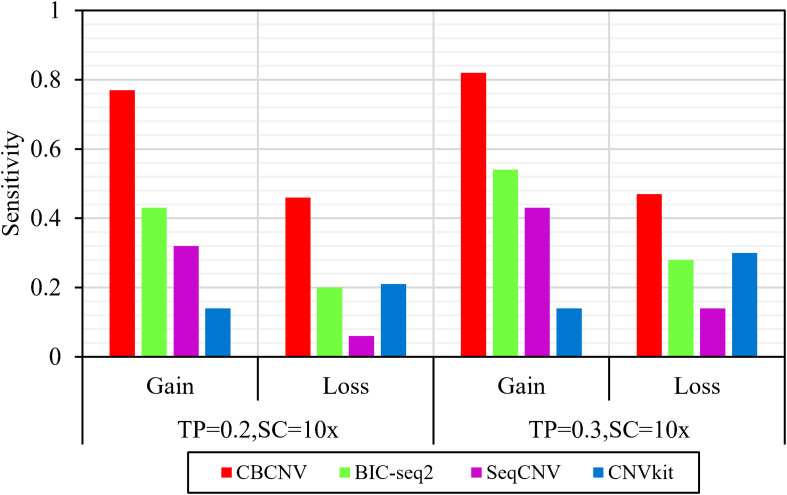
Comparison of the sensitivity of the four methods for detecting gain and loss areas under two sets of simulation settings.

**FIGURE 5:**
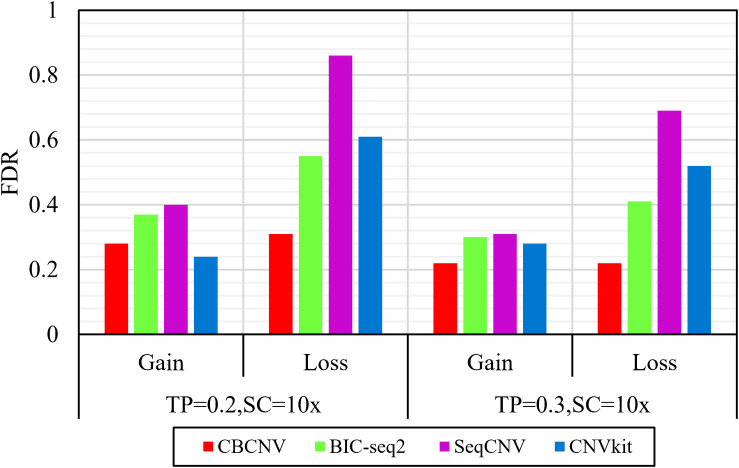
Comparison of the FDR of each method for detecting gain and loss areas under two sets of simulation samples.

Based on the simulated data sets, sensitivity and FDR were considered to analyze and evaluate the performances of the compared methods (CBCNV, BIC-seq2, SeqCNV, and CNVkit) in the gain and loss areas, and the averages of the two indicators were calculated across the 50 samples under different setting conditions. In general, the sensitivity of each method was found to increase with the increase in tumor purity, which demonstrates that the performance of each method was very sensitive to tumor purity. Most methods detected the CNV gain areas more sensitively than the CNV loss areas. Figure 4 describes the sensitivity of each method to the detection of the gain and loss areas under two different sets of conditions. In each set of conditions, CBCNV achieved the highest sensitivity in the gain and loss areas. BIC-seq2 achieved better sensitivity in the gain areas than the other two methods (SeqCNV and CNVkit), and its sensitivity in the loss areas ranked third. The sensitivity of SeqCNV to the detection of the gain areas was between those of BIC-seq2 and CNVkit, and it was insensitive to the detection of the loss areas as compared to the other three methods. CNVkit achieved the lowest sensitivity in the gain areas, but its sensitivity ranked second in the loss areas, which indicates that it is suitable for detecting loss areas. Figure 5 describes the FDR of each method in the detection of gain and loss areas under two different sets of conditions. When tumor purity was equal to 0.2 and 0.3, the FDR of CBCNV ranked second and first in the gain areas, and ranked first in the loss areas. The FDR of BIC-seq2 ranked third and second in the gain and loss areas, respectively. SeqCNV exhibited the largest FDRs in the gain and loss areas, which demonstrates that there were many false positives of the detected CNVs. CNVkit had a medium FDR between those of BIC-seq2 and SeqCNV in the loss areas. In the gain areas, the FDR of CNVkit ranked first when tumor purity = 0.2, and second when tumor purity = 0.3. CNVkit detected the gain areas more accurately than the loss areas. This demonstrates that the DOC-based method detected the gain areas more sensitively than the loss areas. In summary, CBCNV achieved the best tradeoff between sensitivity and FDR in the detection of gain and loss areas.

**FIGURE 6:**
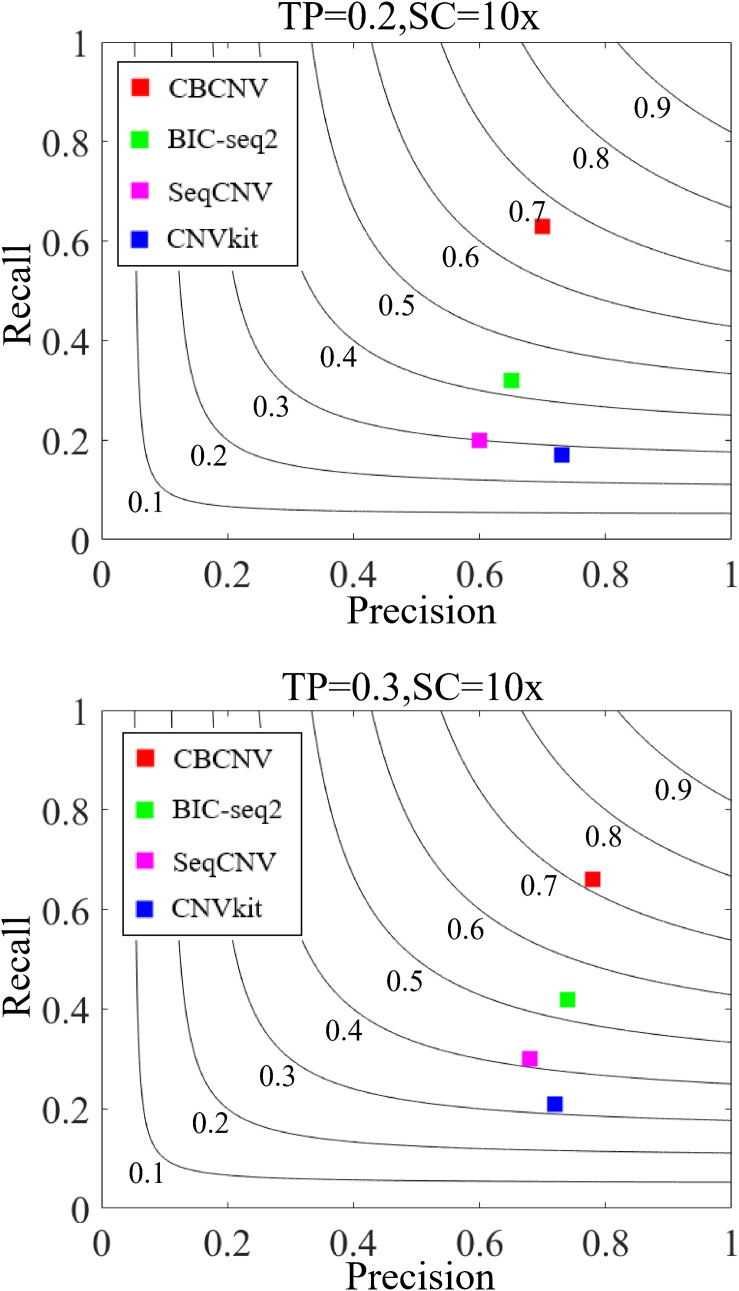
The performance comparison of the four methods in terms of recall, precision and F1-score under two sets of simulation settings. Black curves indicate F1-score levels, which ranges from 0.1 to 0.9. The equations on the left and right sides of the comma represent the tumor purity (TP) and sequencing coverage (SC), respectively.

Three indicators (recall, precision, and F1-score) were adopted to comprehensively evaluate the performance of each method. Recall is defined as the number of correctly detected CNVs divided by the total number of simulated CNVs (Magi et al., 2013). Precision is defined as the number of correctly detected CNVs divided by the total number of detected CNVs (Magi et al., 2013). The F1-score represents the harmonic mean of precision and recall. The three performance indicators are reported as the averages of 50 samples under each set of conditions. Figure 6 detail the F1-score level of each method, from which it is evident that CBCNV achieved the highest recall, followed by BIC-seq2, SeqCNV, and CNVkit. When tumor purity = 0.3, CBCNV got the best precision rate among all methods. When tumor purity = 0.2, CNVkit performed the best in terms of precision, followed by CBCNV, BIC-seq2, and SeqCNV. Moreover, CBCNV achieved the best tradeoff between precision and recall, followed by BIC-seq2, SeqCNV, and CNVkit, which is consistent with the above experimental results.

### Detection of Copy Number Variants From Breast Cancer Samples

To analyze and verify the performance of the proposed method, it was applied to detect four paired whole-genome breast cancer samples (PD4088a, PD4088b, PD4192a, and PD4192b), the details of which were sourced from https://www.ebi.ac.uk/ega/studies under accession EGAS00001000170 (Li Y. Y. et al., 2019). CBCNV was used to detect 22 autosomes in each set of samples, and two well-known methods (BIC-seq2 and CNVkit) were selected for comparison. For a fair comparison, the default parameters were used for these methods to detect the samples. The number of overlapping events and predicted events were used for performance measurement to effectively analyze the advantages and disadvantages of each method. The ground truth file could not be provided in the real data experiment. The number of overlapping events represent the average number of overlapping events for one method and other methods, and number of predicted events represents the total number of events predicted by a method. Table 1 presents the number of overlapping events and predicted events of each method in the 22 autosomes of samples PD4088a and PD4192a, respectively. In the sample PD4088a, it is evident that CBCNV achieved the greatest number of overlapping events and predicted events. BIC-seq2 detects the least number of overlapping events and predicted events, which shows that it is more conservative than the other two methods. CNVkit achieved number of overlapping events and predicted events between CBCNV and BIC-seq2. In the sample PD4192a, CNVkit called a large number of CNV events, but obtained number of overlapping events as many as CBCNV, which means it has detected a large number of non-overlapping events. It indirectly shows that the CNVs detected by CNVkit are likely to contain a large number of false positive events. A small number of overlapping events and predicted events were found by BIC-seq2, performance of which is consistent with the above sample. The number of events detected by CBCNV is between the other two comparison methods, but it obtains the most overlapping events, which fully shows that most of the CNV events detected by CBCNV are true positive events.

**TABLE 1:** Comparison of number of overlapping events (NOE) and predicted events (NPE) for each method on two sets of real samples.

Sample	Indicator	CBCNV	BIC-seq2	CNVkit
PD4088a	NOE	80	19	49
	NPE	510	85	194
PD4192a	NOE	126	20	126
	NPE	482	83	2,156

In order to further verify the performance of each method, we adopt the evaluation method of overlapping density score (ODS) (Yuan et al., 2018), which is defined by the following equation.

(14) $$ODS=O_{m}\cdot O_{r},$$

Where *O*_*m*_ represents the mean number of overlapping events between one method and other comparison methods, *O*_*r*_ represents *O*_*m*_ divided by the total number of CNV events detected by the method. Here, we use Eq. (14) to calculate ODS for each method, and the comparison results are recorded in Table 2. From the experimental results, CBCNV achieve the best ODS in the all samples. ODS of BIC-seq2 are the lowest among all methods. Compared with the above two methods, CNVkit obtain the medium ODS in each group of samples. Overall, CBCNV achieved the best balance between *O*_*m*_ and *O*_*r*_ as compared to the other two methods.

**TABLE 2:** Comparison of ODS for each method on two sets of real samples.

Sample	CBCNV	BIC-seq2	CNVkit
PD4088a	19	6	18
PD4192a	43	7	32

On the basis of the above experiments, we used the catalog of somatic mutations in cancer (COSMIC) database to analyze the biological significance of the detected CNVs. From two pairs of matched breast cancer samples, we found that some of the detected CNVs contained some genes that were related to breast cancer, such as PDZK1 (Kim et al., 2013), XRCC4 (Allen-Brady et al., 2006), Fbxl17 (Mason et al., 2020), ITGBL1 (Li et al., 2015), RORA (Taheri et al., 2017), BAGE (Fujie et al., 1997), AMOTL1 (Couderc et al., 2016), RAP80 (Osorio et al., 2009), PIWIL4 (Wang et al., 2016), CSE1L (Behrens et al., 2001), and USP18 (Tan et al., 2018).

### Evaluation of Running Time

Running time is a critical evaluation indicator to evaluate the performance of the methods. For this, CBCNV and the other three methods (BIC-seq2, SeqCNV, and CNVkit) are tested on 50 simulation samples, which are run on a personal computer with Intel(R) Core (TM) i7-4710MQ CPU @ 2.50 GHz and 16.0 GB memory. The running time of each method is counted as the averages of 50 simulation samples. As shown in Table 3, BIC-seq2 performed the best in terms of running time, followed by CBCNV, CNVkit, and SeqCNV, which shows that CBCNV is a relatively efficient CNV detection tool.

**TABLE 3:** Comparison of running time for each method.

Indicator	CBCNV	BIC-seq2	SeqCNV	CNVkit
Running time (s)	39	8	500	182

## Discussion and Conclusion

In this work, the proposed CBCNV method was developed based on DOC profiles to detect CNVs using next-generation sequencing data, and is suitable for the detection of tumor-normal matched samples. CBCNV uses a local perspective to capture abnormal read depth signals, which are considered to be only a small portion of the overall signals. Its detection concept is different from those of traditional CNV detection methods, which generally construct a statistical model by fitting the read depth signals, then select a reasonable baseline to identify CNVs. Instead, in CBCNV, a clustering algorithm is performed on the read depth segment profile to form clusters of different scales. According to the scales of the clusters, large and small clusters are defined. If a read depth segment originates from a large cluster, its abnormal score is defined as the distance between the read depth segment and the cluster center. If a read depth segment belongs to a small cluster, its abnormal score is defined as the distance between the read depth segment and the center of the closest large cluster. In this way, an abnormal score is assigned to each read depth segment. Based on the abnormal score profile, Tukey’s fences method is adopted to predict CNVs (Zijlstra et al., 2007).

Via the analysis of the concepts of the proposed method, the following characteristics are summarized. (1) CBCNV extracts two features of read depth signals, which fully considers the copy number of each read depth segment and the difference in the ratios of adjacent read depth segments. (2) The traditional outlier detection algorithm was effectively converted to detect CNVs. CBCNV uses a local perspective to identify CNVs, which can objectively reflect the actual state of abnormal read depth signals. It does not require the fitting of the distribution of read depth signals, and cluster-based abnormal scores are constructed for each read depth segment signal to effectively identify the copy number status of adjacent read depth signals. (3) Based on the abnormal score of each read depth segment, Tukey’s fences method is applied to identify CNVs, which does not require the evaluation of the distribution of abnormal scores.

Simulated data sets were used to evaluate the performance of CBCNV, and three popular algorithms were selected for comparison. First, the sensitivity and FDR of each method for the detection of CNVs of different lengths and in gain and loss regions were analyzed and discussed. Via the analysis of the experimental results, it was found that CBCNV achieved the best tradeoff between sensitivity and FDR. Second, three performance indicators (recall, precision, and F1-score) were adopted to comprehensively evaluate the performance of each method. The experimental results proved that CBCNV achieved the best performance in terms of all three indicators. In real data applications, two sets of whole-genome data were used to evaluate the effectiveness of the proposed method. The experimental results demonstrated that CBCNV achieved the best number of overlapping events and overlapping density scores compared to the other two methods in each group of samples. In summary, CBCNV is an effective and reliable CNV detection tool for using on tumor-normal matched samples.

Some shortcomings of the proposed method were also discovered. For example, the selection of the number of clusters (k) is a very important step that may affect the accuracy of the results. In most application scenarios, the performance of the proposed method was superior under this set of parameter settings, which meets the needs of users in most cases. However, in some unique cases, the performance of this set of parameters may not be suitable. In future research, the data size and characteristics will be fully considered to automatically set the parameter k. In addition, in the present study, only two features of read depth were extracted as the input. In future research, multiple factors of read depth signals will be considered to improve the accuracy of the proposed method. Ultimately, CBCNV will be further expanded (Mao et al., 2021) and proved to effectively detect other types of structural variation in multiple application scenarios.

## Data Availability Statement

The datasets presented in this study can be found in online repositories. The names of the repository/repositories and accession number(s) can be found below: https://www.ebi.ac.uk/ega/studies, EGAS00001000170.

## Author Contributions

GL participated in the design of the algorithms and the experiments. JZ participated in the design of the entire framework of CNV detection and directed the whole work, and helped to revise the manuscript. Both authors read the final manuscript and agreed on its contents for submission.

## Conflict of Interest

The authors declare that the research was conducted in the absence of any commercial or financial relationships that could be construed as a potential conflict of interest.
