# Supplementary material for: A Cluster-Based Approach for the Discovery of Copy Number Variations From Next-Generation Sequencing Data
Source: Front Genet. 2021 Jun 28;12:699510. doi: 10.3389/fgene.2021.699510 (PMC8273656; doi:10.3389/fgene.2021.699510)
Supplement: Supplementary file 1 [file Table_1.DOCX]

Supplementary Material

# Supplementary Table 1. Analysis of sensitivity and precision of binsize on 50 simulation samples.

| Binsize(bp) | 1000 | 2000 | 3000 | 4000 | 5000 |
| --- | --- | --- | --- | --- | --- |
| Sensitivity | 0.69 | 0.63 | 0.5 | 0.47 | 0.43 |
| Precision | 0.41 | 0.7 | 0.75 | 0.78 | 0.74 |

# Supplementary Table 2. Analysis of sensitivity and precision of number of clusters on 50 simulation samples.

| k | 3 | 5 | 7 | 10 |
| --- | --- | --- | --- | --- |
| Sensitivity | 0.61 | 0.63 | 0.64 | 0.59 |
| Precision | 0.73 | 0.7 | 0.71 | 0.67 |

**Supplementary Table 3.** Analysis of sensitivity and precision of number of neighbors on 50 simulation samples.

| L | 5 | 10 | 15 | 20 |
| --- | --- | --- | --- | --- |
| Sensitivity | 0.62 | 0.63 | 0.63 | 0.61 |
| Precision | 0.68 | 0.7 | 0.72 | 0.71 |

**Supplementary Table 4.** Analysis of sensitivity and precision of abnormal weight on 50 simulation samples.

| w | 1.5 | 3 |
| --- | --- | --- |
| Sensitivity | 0.63 | 0.53 |
| Precision | 0.7 | 0.82 |

**Supplementary Table 5.** Analysis of the sensitivity of three methods at the six CNV length levels under 50 simulation samples.

| CNV length (kb) | CBCNV | BreakDancer | TIDDIT |
| --- | --- | --- | --- |
| 2 | 0.47 | 0.49 | 0.56 |
| 6 | 0.47 | 0.42 | 0.51 |
| 10 | 0.67 | 0.38 | 0.48 |
| 30 | 0.62 | 0.19 | 0.2 |
| 50 | 0.72 | 0.15 | 0.09 |
| 100 | 0.75 | 0.1 | 0.06 |

**Supplementary Table 6.** Analysis of the false discovery rate (FDR) of three methods at the six CNV length levels under 50 simulation samples.

| Indicator | CBCNV | BreakDancer | TIDDIT |
| --- | --- | --- | --- |
| FDR | 0.3 | 0.8 | 0.67 |

**Supplementary Table 7.** Analysis of recall, precision, F1-score of three methods on 50 simulation samples.

| Method | Recall | Precision | F1-score |
| --- | --- | --- | --- |
| CBCNV | 0.63 | 0.7 | 0.66 |
| BreakDancer | 0.27 | 0.2 | 0.23 |
| TIDDIT | 0.28 | 0.33 | 0.31 |
